# Erysipelas Complicated by COVID‐19

**DOI:** 10.1111/srt.70062

**Published:** 2024-10-04

**Authors:** Jie‐Lun Wen, Qi‐Ze Wang, Ming‐Hui Peng, Lu Liu, Li‐Yue Sun

**Affiliations:** ^1^ School of medicine Jinan University Guangzhou Guangdong China; ^2^ Department of Health Management Centre Zhongshan Hospital Fudan University Shanghai Shanghai China; ^3^ Department of General Practice Zhongshan Hospital Fudan University Shanghai Shanghai China; ^4^ Department of Dermatology First Affiliated Hospital Anhui Medical University Hefei Anhui China

Dear Editor,

A 34‐year‐old male clinical doctor developed erysipelas during two separate coronavirus disease 2019 (COVID‐19) infections, having not received any vaccinations.

In December 2022, during his first infection, the erysipelas manifested on the front of his left sole, primarily affecting the middle and anterior regions and covering a substantial area. The skin displayed intense, dark red erythema, indicative of severe local inflammation (Figure 1A). This erythema was sharply demarcated, clearly distinguishing it from the surrounding healthy skin. Additionally, the lesion was associated with an increase in local skin temperature and a burning sensation while walking. During his second COVID‐19 infection, in May 2023, the erysipelas primarily affected the front of the left dorsum, especially around the base and between the second and fifth toes. In this instance, the skin showed mild, localized erythema that was lighter in color and covered a smaller area, suggesting a less severe degree of inflammation. The erythema had relatively distinct borders, contrasting sharply with the surrounding normal skin (Figure 1B). The affected area was irregularly shaped, extending over the bases of the toes and into some interdigital spaces. The erythematous region appeared slightly swollen with a subtle sheen, possibly indicating mild edema. Compared to the first infection, the erysipelas in the second instance was less extensive, milder, and less painful. Both episodes resolved within a week following treatment with amoxicillin.

**FIGURE 1:**
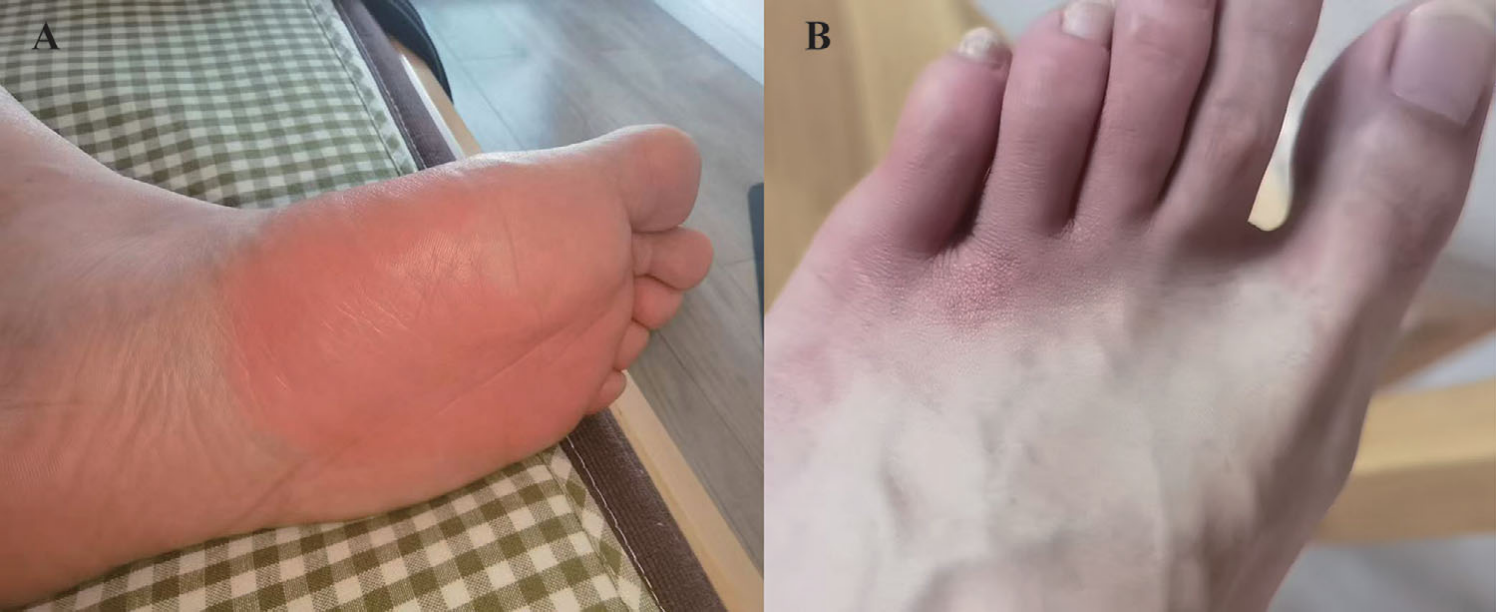
(A) First occurrence of erysipelas complicated by COVID‐19 in December 2022; (B) second occurrence of erysipelas complicated by COVID‐19 in May 2023.

Erysipelas is a superficial infection of the skin and soft tissue caused by Group A Streptococcus, commonly affecting the lower limbs and face. This report presents a case of erysipelas occurring during two separate COVID‐19 infections. COVID‐19 can not only weaken the immune system, but it can also heighten susceptibility to bacterial infections. As a result of this weakened immunity, the risk of infection is further increased during extended hospitalizations [1]. Data from previous studies indicate that 0.19%–20.45% of COVID‐19 patients may develop skin symptoms, such as rashes or other skin abnormalities. However, these dermatological reactions are relatively rare in Asian populations compared to other regions [2]. Recent research has reported a case of recurrent erysipelas in a patient who contracted COVID‐19 during treatment, suggesting a possible link between COVID‐19 and skin infections [3]. Understanding this connection is crucial, especially given the potential new risks associated with the widespread use of alcohol‐based hand sanitizers and disinfectants during the pandemic [4].

## Ethics Statement

The study was conducted in accordance with the Declaration of Helsinki and obtained informed consent from the patient.

## Conflicts of Interest

The authors declare no conflicts of interest.

## Data Availability

The data that support the findings of this study are available from the corresponding author upon reasonable request.
